# Supplementary material for: Time-series transcriptome analysis identified differentially expressed genes in broiler chicken infected with mixed Eimeria species
Source: Front Genet. 2022 Aug 8;13:886781. doi: 10.3389/fgene.2022.886781 (PMC9393255; doi:10.3389/fgene.2022.886781)
Supplement: Supplementary file 2 [file DataSheet1.ZIP › 7dpi_KEGG.Gsea.1625115961950/KEGG_NATURAL_KILLER_CELL_MEDIATED_CYTOTOXICITY.html]

Details for gene set KEGG\_NATURAL\_KILLER\_CELL\_MEDIATED\_CYTOTOXICITY[GSEA]

|  || Dataset | TMM\_7dpi\_gct\_format\_7dpi\_gct\_format.Class\_4dpi.cls #PC\_versus\_NC.Class\_4dpi.cls #PC\_versus\_NC\_repos |
| Phenotype | Class\_4dpi.cls#PC\_versus\_NC\_repos |
| Upregulated in class | 0 |
| GeneSet | KEGG\_NATURAL\_KILLER\_CELL\_MEDIATED\_CYTOTOXICITY |
| Enrichment Score (ES) | -0.54613936 |
| Normalized Enrichment Score (NES) | -1.8778607 |
| Nominal p-value | 0.0 |
| FDR q-value | 0.01767293 |
| FWER p-Value | 0.0206 |
Table: GSEA Results Summary

  

Fig 1: Enrichment plot: KEGG\_NATURAL\_KILLER\_CELL\_MEDIATED\_CYTOTOXICITY      
 Profile of the Running ES Score & Positions of GeneSet Members on the Rank Ordered List

  

| SYMBOL | TITLE | RANK IN GENE LIST | RANK METRIC SCORE | RUNNING ES | CORE ENRICHMENT || 1 | CHP1 | na | 1292 | 0.430 | -0.0977 | No |
| 2 | HRAS | na | 1342 | 0.420 | -0.0916 | No |
| 3 | VAV3 | na | 1390 | 0.408 | -0.0857 | No |
| 4 | BID | na | 1694 | 0.334 | -0.1029 | No |
| 5 | SHC4 | na | 2866 | 0.143 | -0.1975 | No |
| 6 | RAC1 | na | 2998 | 0.126 | -0.2054 | No |
| 7 | TNFRSF10B | na | 3036 | 0.122 | -0.2055 | No |
| 8 | PPP3R1 | na | 3345 | 0.081 | -0.2294 | No |
| 9 | MAP2K2 | na | 3435 | 0.070 | -0.2351 | No |
| 10 | PPP3CA | na | 4410 | -0.040 | -0.3157 | No |
| 11 | MICA | na | 4665 | -0.066 | -0.3353 | No |
| 12 | RAF1 | na | 5142 | -0.116 | -0.3724 | No |
| 13 | MAP2K1 | na | 5188 | -0.120 | -0.3732 | No |
| 14 | SHC2 | na | 5501 | -0.149 | -0.3957 | No |
| 15 | SHC1 | na | 5796 | -0.179 | -0.4160 | No |
| 16 | SH2D1A | na | 5807 | -0.180 | -0.4125 | No |
| 17 | RAC3 | na | 6076 | -0.210 | -0.4298 | No |
| 18 | PLCG1 | na | 6241 | -0.228 | -0.4380 | No |
| 19 | IFNAR2 | na | 6336 | -0.238 | -0.4401 | No |
| 20 | CASP3 | na | 6448 | -0.248 | -0.4434 | No |
| 21 | GRB2 | na | 6686 | -0.274 | -0.4566 | No |
| 22 | FASLG | na | 6950 | -0.302 | -0.4713 | No |
| 23 | PTK2B | na | 7166 | -0.325 | -0.4814 | No |
| 24 | KRAS | na | 7206 | -0.330 | -0.4766 | No |
| 25 | NRAS | na | 7474 | -0.358 | -0.4903 | No |
| 26 | IFNGR2 | na | 7611 | -0.373 | -0.4926 | No |
| 27 | RAC2 | na | 7904 | -0.409 | -0.5071 | No |
| 28 | NFAT5 | na | 8371 | -0.468 | -0.5348 | Yes |
| 29 | NFATC1 | na | 8451 | -0.478 | -0.5298 | Yes |
| 30 | PIK3CG | na | 8478 | -0.481 | -0.5203 | Yes |
| 31 | PRKCA | na | 8704 | -0.513 | -0.5267 | Yes |
| 32 | SOS1 | na | 8869 | -0.533 | -0.5275 | Yes |
| 33 | PAK1 | na | 8943 | -0.541 | -0.5205 | Yes |
| 34 | PPP3CB | na | 8981 | -0.547 | -0.5103 | Yes |
| 35 | FCER1G | na | 9176 | -0.578 | -0.5125 | Yes |
| 36 | IFNAR1 | na | 9530 | -0.636 | -0.5267 | Yes |
| 37 | PIK3R2 | na | 9597 | -0.645 | -0.5165 | Yes |
| 38 | ZAP70 | na | 9604 | -0.646 | -0.5014 | Yes |
| 39 | SYK | na | 9655 | -0.655 | -0.4897 | Yes |
| 40 | PTPN6 | na | 9763 | -0.668 | -0.4824 | Yes |
| 41 | PRKCB | na | 9867 | -0.685 | -0.4744 | Yes |
| 42 | TNFSF10 | na | 10126 | -0.736 | -0.4781 | Yes |
| 43 | MAPK3 | na | 10320 | -0.774 | -0.4755 | Yes |
| 44 | PIK3CB | na | 10335 | -0.777 | -0.4578 | Yes |
| 45 | LCP2 | na | 10364 | -0.782 | -0.4412 | Yes |
| 46 | SH2D1B | na | 10465 | -0.803 | -0.4301 | Yes |
| 47 | PTPN11 | na | 10560 | -0.822 | -0.4180 | Yes |
| 48 | LCK | na | 10662 | -0.845 | -0.4060 | Yes |
| 49 | PLCG2 | na | 10697 | -0.853 | -0.3881 | Yes |
| 50 | PIK3CD | na | 10707 | -0.855 | -0.3681 | Yes |
| 51 | CD48 | na | 10712 | -0.856 | -0.3476 | Yes |
| 52 | SH3BP2 | na | 10828 | -0.887 | -0.3357 | Yes |
| 53 | PIK3CA | na | 10920 | -0.912 | -0.3212 | Yes |
| 54 | PIK3R5 | na | 10941 | -0.918 | -0.3006 | Yes |
| 55 | PIK3R3 | na | 11045 | -0.949 | -0.2862 | Yes |
| 56 | CD244 | na | 11224 | -1.008 | -0.2767 | Yes |
| 57 | PIK3R1 | na | 11265 | -1.023 | -0.2552 | Yes |
| 58 | SOS2 | na | 11362 | -1.063 | -0.2374 | Yes |
| 59 | FYN | na | 11389 | -1.076 | -0.2135 | Yes |
| 60 | CD247 | na | 11411 | -1.084 | -0.1889 | Yes |
| 61 | NFATC2 | na | 11531 | -1.128 | -0.1715 | Yes |
| 62 | ITGB2 | na | 11693 | -1.235 | -0.1550 | Yes |
| 63 | BRAF | na | 11724 | -1.264 | -0.1269 | Yes |
| 64 | NFATC3 | na | 11833 | -1.382 | -0.1024 | Yes |
| 65 | FAS | na | 11850 | -1.412 | -0.0695 | Yes |
| 66 | IFNGR1 | na | 11917 | -1.547 | -0.0374 | Yes |
| 67 | VAV2 | na | 11979 | -1.860 | 0.0026 | Yes |
Table: GSEA details [plain text format]

  

Fig 2: KEGG\_NATURAL\_KILLER\_CELL\_MEDIATED\_CYTOTOXICITY      
 Blue-Pink O' Gram in the Space of the Analyzed GeneSet

  

Fig 3: KEGG\_NATURAL\_KILLER\_CELL\_MEDIATED\_CYTOTOXICITY: Random ES distribution      
 Gene set null distribution of ES for **KEGG\_NATURAL\_KILLER\_CELL\_MEDIATED\_CYTOTOXICITY**
